# Invisibility and devaluation of nursing work: related factors and coping strategies

**DOI:** 10.17533/udea.iee.v42n1e01

**Published:** 2024-04-11

**Authors:** José Jeová Mourão Netto

**Affiliations:** 1 Assistance nurse at Hospital Estadual Regional Norte, PhD in Clinical Care in Nursing and Health, Professor of the Nursing Course at Luciano Feijão College, Sobral, Brazil. Email: jeovamourao@yahoo.com.br Faculdade Luciano Feijão Luciano Feijão College Sobral Brazil jeovamourao@yahoo.com.br

Nursing represents the largest professional category in the health field worldwide, with 27.9 million professionals, representing 59% of the entire health workforce.^1^The World Health Organization has warned of the need for greater investments in training, better working conditions and encouraging the development of nursing leadership as one of the means to achieve universal health coverage and the Sustainable Development Goals (SDGs).^1^However, even though they are essential to health systems, these professionals face problems related to the representation of their image, which contributes to a scenario of devaluation, which has proved to be an obstacle to the development of the profession. Thus, in an exercise of understanding this phenomenon, it is considered that the factors that follow contribute greatly to configure the devaluation scenario.

## Professionals with weak professional identity and limited knowledge about their work process

The professional identity of nurses is built from several elements, including what society thinks and expresses about them, in terms of their care, educational, scientific, social and political function.^2^Studies have shown that nurses have little clarity about what they do, a fact that, added to the context of increasing precariousness of work, can deepen frustration and little identity with the profession.^3^There are discussions about the need to overcome vague definitions in nursing, as they do not bring the elements that allow differentiating the doing of nurses from the doing of other professionals, which may explain the fact that many nurses are unable to express a definition of nursing, or systematize what their attributions are, and there is an urgent need for clearer concepts that in fact allow nurses to recognize the attributes that distinguish the profession from others.^3^

## Complex work, but unnoticed: incomprehension and invisibility of nursing work

People have only a superficial understanding of the nurse's work, built from what they perceive in the services or have access to in news or film productions, and it is necessary that we promote, from education about what we do, the evolution of understanding to knowledge about our work. Because it is a complex work but little apparent, society tries to understand the nursing work by associating it with what it already knows about health work. Often, this prior knowledge is related to the physician's work, because his work is more consolidated in the social ideal. Perhaps, therefore, the activities that most characterize the work of nursing for society are almost always related to the physician: administration of drugs prescribed by the physician, the physician's assistant, think that nurses have less knowledge and consider that nurses are subordinate to these professionals.

In an attempt to understand this work, it is common that they also associate it with lay care, since feeding, bathing, dressing, changing clothes and sheets are also nursing activities. However, we need to explain that while they look the same, they are very different activities. For example, the way to feed a patient by a nursing professional involves complex knowledge and skills, as we have to consider the consistency of the food, assess the ability to swallow, choose the optimal position, and observe risks and signs of broncho aspiration, possible gastrointestinal reactions and acceptance. The complexity of care may go unnoticed if the professional does not explain to the patient, caregivers and family all the knowledge and skills involved in these activities. Thus, for society to evolve from vague understanding to knowledge about what we do, it will need our mediation. 

Another aspect to be considered is the fact that caring has historically been considered women's work. In this sense, in a still patriarchal society, the lower hierarchical order of society associated with care work continues to perpetuate the social oppression of caregivers who, traditionally, were not paid and, also because of this, related to low socioeconomic status,^4^ developing an essential work, but which ends up being invisible due to its status in society. Nurses need to explore alternative ways of communicating the contributions of their role and the impact on health outcomes and quality of life, as well as making the profession more visible and explicit.^5^

## Misconceptions and stereotypes about work and nursing professionals

Society's lack of knowledge about nursing practice and the consequent invisibility of its work is related to distorted and stereotyped images of these professionals. The inaccurate and distorted images of nursing limit its public understanding, preventing us from being seen as well-informed and qualified health professionals, because, due to the complexity of our work, not all activities are apparent, requiring resources that can help them create a positive image from what we really do and are: leaders, administrators and professionals who provide care with resoluteness and essential to all health systems. 

We observed few strategies to confront stereotypes in the media about nursing, the most frequent being those of heroin, angel, prostitute, seductive woman or servant,^5^ stereotypes repeatedly represented in films and series, feeding the social ideology with mistaken impressions. Even understanding as angels and heroes also does not help in creating an image close to reality, as it creates a perception that skill and knowledge are not so important, believing that the skills to be a good professional are somehow innate or conferred on us and not acquired by study and training, attributing us superhuman characteristics,^5^ which does not benefit us, because both superheroes and angels do not die, they also do not require training or a salary compatible with their attributions; unlike nursing professionals. The weak professional identity added to the difficulty of understanding our work creates a vacuum that is configured as a space for building different ideas, being a fertile field for the establishment of stereotypes. Thus, the more apparent, understandable and clear the nursing work, the less room there will be for this type of construction. 

## The social devaluation

The repercussion of all aspects discussed so far (weak professional identity, ill-defined contours of the profession, invisibility and stereotypes) contribute to the social devaluation of work, contributing to little recognition and prestige and, also for this reason, professional dissatisfaction. A review, with the objective of describing the perception of young people about the work of nursing, identified that the participants relate it to precarious working conditions and limited autonomy, and this work is seen mainly as caring for and helping patients and was considered inferior to the work of the physician. Young people did not mention the knowledge and training of professionals and pointed out the social status of nursing as low.^6^

The low valuation of nurses is reflected in poor working conditions, in the absence of decent salaries, in the lack of an adequate weekly workload compatible with the complexity of this activity, in the inadequacy of places for rest and food in many institutions, low electoral success for those who are willing to try positions in party politics^7^ and in the underrepresentation of this group in decision-making spaces.

## Proposing coping strategies

Recognizing what we do and communicating this to patients is the first step. Society needs to recognize that we develop a different work process from other health professionals, as we are the only professionals who simultaneously: (1) lead people, as we coordinate the work process of nursing assistants and technicians; (2) manage material resources and care processes, as we monitor all therapeutic interventions performed on the patient, which allows us to say that our work directs the work process of other professionals; (3) and provide care on a readiness (when activated) and wakefulness basis (24 hours at the bedside), anchored in dense scientific knowledge. 

During interventions with patients, it is important that nurses use the proper language of nursing, such as nursing diagnoses, interventions and results, to show the scientificity involved in this practice, also causing the patients to distance our care, scientific and professional, from lay care, as well as from the care of other team members. In order to communicate to society about what we do, the use of virtual social networks can constitute a powerful strategy for the dissemination of a more positive and closer image of reality, changing the social idea about the profession.

With regard to society's impression of an alleged subalternity to the figure of the physician, it is necessary that we recognize expressions that contribute to validate these mistaken impressions and avoid them. Expressions such as: "you will talk to the physician, because I am only the nurse", as if the nurse's work had little importance in the health production process; "you will be good soon, because the physician will attend you", reinforcing an idea that the cure depends only on a single professional; "good morning, you will be attended by me, the nurse, and the physician", the expression physician, irretrievably, creates an idea of superiority and hierarchy^8^ in relation to the nurse, configuring a situation of symbolic violence, and it is important to avoid it.

Knowing and reflecting more about what we do is a structuring step to overcome this reality, communicating this to society, as it is not possible to value what is not known or is invisible. This change depends on what each nurse does on a daily basis, on how they communicate with their patients, companions, caregivers and family members in the various practice scenarios.
